# Supplementary material for: Dynamic fMRI networks of human emotion
Source: eLife. 2026 Aug 28;14:RP106070. doi: 10.7554/eLife.106070 (PMC13524378; doi:10.7554/eLife.106070)
Supplement: Peer review history

# Editor report: eLife Assessment

**DOI:** 10.7554/eLife.106070.3.sa0

**Authors:** Xiaoqing Hu

**Affiliations:** University of Hong Kong China

**Keywords:** Convincing, Important

This manuscript provides **important** information on the neurodynamics of emotional processing while participants were watching movie clips. This work provides **convincing** results in deciphering the temporal-spatial dynamics of emotional processing. This work will be of interest to affective neuroscientists and fMRI researchers in general.

# Referee report: Reviewer #1 (Public review)

**DOI:** 10.7554/eLife.106070.3.sa1

Summary and strengths:

In this manuscript, the authors endeavor to capture the dynamics of emotion-related brain networks. They employ slice-based fMRI combined with ICA on fMRI time series recorded while participants viewed a short movie clip. This approach allowed them to track the time course of four non-noise independent components at an effective 2s temporal resolution at the BOLD level. Notably, the authors report a temporal sequence from input to meaning, followed by response, and finally default mode networks, with significant overlap between stages. The use of ICA offers a data-driven method to identify large-scale networks involved in dynamic emotion processing. Overall, this paradigm and analytical strategy mark an important step forward in shifting affective neuroscience toward investigating temporal dynamics rather than relying solely on static network assessments.

(1) One of the main advantages highlighted is the improved temporal resolution offered by slice-based fMRI. However, the manuscript does not clearly explain how this method achieves a higher effective resolution, especially since the results still show a 2s temporal resolution-comparable to conventional methods. Clarification on this point would help readers understand the true benefit of the approach.

(2) While combining ICA with task fMRI is an innovative approach to study the spatiotemporal dynamics of emotion processing, task fMRI typically relies on modeling the hemodynamic response (e.g., using FIR or IR models) to mitigate noise and collinearity across adjacent trials. The current analysis uses unmodeled BOLD time series, which might risk suffering from these issues.

(3) The study's claims about emotion dynamics are derived from fMRI data, which are inherently affected by the hemodynamic delay. This delay means that the observed time courses may differ substantially from those obtained through electrophysiology or MEG studies. A discussion on how these fMRI-derived dynamics relate to-or complement-is critical for the field to understand the emotion dynamics.

(4) Although using ICA to differentiate emotion elements is a convenient approach to tell a story, it may also be misleading. For instance, the observed delayed onset and peak latency of the 'response network' might imply that emotional responses occur much later than other stages, which contradicts many established emotion theories. Given the involvement of large-scale brain regions in this network, the underlying reasons for this delay could be very complex.

Added after revision: In the response letter, the authors have provided clear responses to these comments and improved the manuscript.

# Author comment: Author response

**DOI:** 10.7554/eLife.106070.3.sa2

**Authors:** Niels Janssen, Uriel KA Elvira, Joost Janssen, Theo van Erp

**Affiliations:** Universidad de La Laguna La Laguna Spain; Universidad de La Laguna La Laguna Spain; Hospital General Universitario Gregorio Marañón Madrid Spain; University of California, Irvine Irvine United States

The following is the authors’ response to the original reviews.

**Reviewer #1 (Public review):**

Summary:

In this manuscript, the authors endeavor to capture the dynamics of emotion-related brain networks. They employ slice-based fMRI combined with ICA on fMRI time series recorded while participants viewed a short movie clip. This approach allowed them to track the time course of four non-noise independent components at an effective 2s temporal resolution at the BOLD level. Notably, the authors report a temporal sequence from input to meaning, followed by response, and finally default mode networks, with significant overlap between stages. The use of ICA offers a data-driven method to identify large-scale networks involved in dynamic emotion processing. Overall, this paradigm and analytical strategy mark an important step forward in shifting affective neuroscience toward investigating temporal dynamics rather than relying solely on static network assessments

Strengths:

(1) One of the main advantages highlighted is the improved temporal resolution offered by slice-based fMRI. However, the manuscript does not clearly explain how this method achieves a higher effective resolution, especially since the results still show a 2s temporal resolution, comparable to conventional methods. Clarification on this point would help readers understand the true benefit of the approach.

(2) While combining ICA with task fMRI is an innovative approach to study the spatiotemporaldynamics of emotion processing, task fMRI typically relies on modeling the hemodynamic response (e.g., using FIR or IR models) to mitigate noise and collinearity across adjacent trials. The current analysis uses unmodeled BOLD time series, which might risk suffering from these issues.

(3) The study's claims about emotion dynamics are derived from fMRI data, which are inherently affected by the hemodynamic delay. This delay means that the observed time courses may differ substantially from those obtained through electrophysiology or MEG studies. A discussion on how these fMRI-derived dynamics relate to - or complement - is critical for the field to understand the emotion dynamics.

(4) Although using ICA to differentiate emotion elements is a convenient approach to tell a story, it may also be misleading. For instance, the observed delayed onset and peak latency of the 'response network' might imply that emotional responses occur much later than other stages, which contradicts many established emotion theories. Given the involvement of largescale brain regions in this network, the underlying reasons for this delay could be very complex.

Concerns and suggestions:

However, I have several concerns regarding the specific presentation of temporal dynamics in the current manuscript and offer the following suggestions.

(1) One selling point of this work regarding the advantages of testing temporal dynamics is the application of slice-based fMRI, which, in theory, should improve the temporal resolution of the fMRI time course. Improving fMRI temporal resolution is critical for a research project on this topic. The authors present a detailed schematic figure (Figure 2) to help readers understand it. However, I have difficulty understanding the benefits of this method in terms of temporal resolution.

(a) In Figure 2A, if we examine a specific voxel in slice 2, the slice acquisitions occur at 0.7s, 2.7s, and 4.7s, which implies a temporal resolution of 2s rather than 0.7s. I am unclear on how the temporal resolution could be 0.7s for this specific voxel. I would prefer that the authors clarify this point further, as it would benefit readers who are not familiar with this technology.

We very much appreciate these concerns as they highlight shortcomings in our explanation of the method. Please note that the main explanation of the method (and comparison with expected HRF and FIR based methods) is done in Janssen et al. (2018, NeuroImage; see further explanations in Janssen et al., 2020). However, to make the current paper more selfcontained, we provided further explanation of the Slice-Based method in Figure 2. With respect to the specific concern of the reviewer, in the hypothetical example used in Figure 2, the temporal resolution of the voxel on slice 2 is 0.7s because it combines the acquisitions from stimulus presentations across all trials. Specifically, given the specific study parameters as outlined in Figures 2A and B, slice 2 samples the state of the brain exactly 0s after stimulus presentation on trial 1 (red color), 0.7s after stimulus presentation on trial 3 (green color), and 1.3s after stimulus presentation on trial 2 (yellow color). Thus after combining data acquisitions across these three 3 stimuli presentations, slice 2 has sampled the state of the brain at timepoints that are multiples of 0.7s starting from stimulus onset. This is why we say that the theoretical maximum temporal resolution is equal to the TR divided by the number of slices (in the example 2/3 = 0.7s, in the actual experiment 3/39 = 0.08s). In the current study we used temporal binning across timepoints to reduce the temporal resolution (to 2 seconds) and improve the tSNR.

We have updated the legend of Figure 3 to more clearly explain this issue.

(b) Even with the claim of an increased temporal resolution (0.7s), the actual data (Figure 3) still appears to have a 2s resolution. I wonder what specific benefit slice-based fMRI brings in terms of testing temporal dynamics, aside from correcting the temporal distortions that conventional fMRI exhibits.

This is a good point. In the current experiment, the TR was 3s, but we extracted the fMRI signal at 2s temporal resolution, which means an increment of 33%. In this study we did not directly compare the impact of different temporal resolutions on the efficacy of detection of network dynamics. Indeed, we agree with the reviewer that there remain many unanswered questions about the issue of temporal resolution of the extracted fMRI signal and the impact on the ability to detect fMRI network dynamics. We think that questions such as those posed by the reviewer should be addressed in future studies that are directly focused on this issue. We have updated our discussion section (page 21-22) to more clearly reflect this point of view.

(2) In task-fMRI, the hemodynamic response is usually estimated using a specific model (e.g., FIR, IR model; see Lindquist et al., 2009). These models are effective at reducing noise and collinearity across adjacent trials. The current method appears to be conducted on unmodeled BOLD time series.

(a) I am wondering how the authors avoid the issues that are typically addressed by these HRF modeling approaches. For example, if we examine the baseline period (say, -4 to 0s relative to stimulus onset), the activation of most networks does not remain around zero, which could be due to delayed influences from the previous trial. This suggests that the current time course may not be completely accurate.

We thank the reviewer for highlighting this issue. Let us start by reiterating what we stated above: That there are many issues related to BOLD signal extraction and fMRI network discovery in task-based fMRI that remain poorly understood and should be addressed in future work. Such work should explore, for example, the impact of using a FIR vs Slice-based method on the discovery of networks in task-fMRI. These studies should also investigate the impact of different types of baselines and baseline durations on the extraction of the BOLD signal and network discovery. For the present purposes, our goal was not to introduce a new technique of fMRI signal extraction, but to show that the slice-based technique, in combination with ICA, can be used to study the brain’s networks dynamics in an emotional task. In other words, while we clearly appreciate the reviewer’s concerns and have several other studies underway that directly address these concerns, we believe that such concerns are better addressed in independent research. See our discussion on page 21-22 that addresses this issue.

(b) A related question: if the authors take the spatial map of a certain network and apply a modeling approach to estimate a time series within that network, would the results be similar to the current ICA time series?

Interesting point. Typically in a modeling approach the expected HRF (e.g., the double gamma function) is fitted to the fMRI data. Importantly, this approach produces static maps of the fit between the expected HRF and the data. By contrast, model-free approaches such as FIR or slice-based methods extract the fMRI signal directly from the data without making apriori assumptions about the expected shape of the signal. These approaches do not produce static maps but instead are capable of extracting the whole-brain dynamics during the execution of a task (event-related dynamics). These data-driven approaches (FIR, SliceBased, etc) are therefore a necessary first step in the analyses of the dynamics of brain activity during a task. The subsequent step involves the analyses of these complex eventrelated brain dynamics. In the current paper we suggest that a straightforward way to do this is to use ICA which produces spatial maps of voxels with similar time courses, and hence, yields insights into the temporal dynamics of whole-brain fMRI networks. As we mentioned above, combining ICA with a high temporal resolution data-driven signal is new and there are many new avenues for research in this burgeoning new field.

(3) Human emotion should be inherently fast to ensure survival, as shown in many electrophysiology and MEG studies. For example, the dynamics of a fearful face can occur within 100ms in subcortical regions (Méndez-Bértolo et al., 2016), and general valence and arousal effects can occur as early as 200ms (e.g., Grootswagers et al., 2020; Bo et al., 2022). In contrast, the time-to-peak or onset timing in the BOLD time series spans a much larger time range due to the hemodynamic delay. fMRI findings indeed add spatial precision to our understanding of the temporal dynamics of emotion, but could the authors comment on how the current temporal dynamics supplement those electrophysiology studies that operate on much finer temporal scales?

We really like this point. One way that EEG and fMRI are typically discussed is that these two approaches are said to be complementary. While EEG is able to provide information on temporal dynamics, but not spatial localization of brain activity, fMRI cannot provide information on the temporal dynamics, but can provide insights into spatial localization. Our study most directly challenges the latter part of this statement. We believe that by using tasks that highlight “slow” cognition, fMRI can be used to reveal not only spatial but also temporal information of brain activity. The movie task that we used presumably relies on a kind of “slow” cognition that takes place on longer time scales (e.g., the construction of the meaning of the scene). Our results show that with such tasks, whole-brain networks with different temporal dynamics can be separated by ICA, at odds with the claim that fMRI is only good for spatial information. One avenue of future research would be to attempt such “slow” tasks directly with EEG and try to find the electrical correlates of the networks detected in the current study.

We hope to have answered the concerns of the reviewer.

(4) The response network shows activation as late as 15 to 20s, which is surprising. Could the authors discuss further why it takes so long for participants to generate an emotional response in the brain?

We thank the reviewer for this question. Our study design was such that there was an initial movie clip that lasted 12.5s, which was then followed by a two-alternative forced-choice decision task (including a button press, 2.5s), and finally followed by a 10s rest period. We extracted the fMRI signal across this entire 25s period (actually 28s because we also took into account some uncertainty in BOLD signal duration). Network discovery using ICA then showed various networks with distinct time courses (across the 25s period), including one network (IC2 response) that showed a peak around 21s (see Figure 3). Given the properties of the spatial map (eg., activity in primary motor areas, Figure 4), as well as the temporal properties of its timecourse (e.g., peak close to the response stage of the task), we interpreted this network as related to generating the manual response in the two-alternative forced-choice decision task. Further analyses showed that this aspect of the task (e.g., deciding the emotion of the character in the movie clip) was also sensitive to the emotional content of the earlier movie clip (Figure 6 and 7).

We have further clarified this aspect of our results (see pages 16-17). We thank the reviewer for pointing this out.

(5) Related to 4. In many theories, the emotion processing stages-including perception, valuation, and response-are usually considered iterative processes (e.g., Gross, 2015), especially in real-world scenarios. The advantage of the current paradigm is that it incorporates more dynamic elements of emotional stimuli and is closer to reality. Therefore, one might expect some degree of dynamic fluctuation within the tested brain networks to reflect those potential iterative processes (input, meaning, response). However, we still do not observe much brain dynamics in the data. In Figure 5, after the initial onset, most network activations remain sustained for an extended period of time. Does this suggest that emotion processing is less dynamic in the brain than we thought, or could it be related to limitations in temporal resolution? It could also be that the dynamics of each individual trial differ, and averaging them eliminates these variations. I would like to hear the authors' comments on this topic.

We thank the reviewer for this interesting question. We are assuming the reviewer is referring to Figure 3 and not Figure 5. Indeed what Figure 3 shows is the average time course of each detected network across all subjects and trial types. This figure therefore does not directly show the difference in dynamics between the different emotions. However, as we show in further analyses that examine how emotion modulates specific aspects of the fMRI signal dynamics (time to peak, peak value, duration) of different networks, there are differences in the dynamics of these networks depending on the emotion (Figure 6 and 7). Thus, our results show that different emotions evoked by movie clips differ in their dynamics. Obviously, generalizing this to say that in general, different emotions have different brain dynamics is not straightforward and would require further study (probably using other tasks, and other emotions). We have updated the discussion section as well as the caption of Figure 3 to better explain this issue (see also comments by reviewer 2).

(6) The activation of the default mode network (DMN), although relatively late, is very interesting. Generally, one would expect a deactivation of this network during ongoing external stimulation. Could this suggest that participants are mind-wandering during the later portion of the task?

Very good point. Indeed this is in line with our interpretation. The late activity of the default mode network could reflect some further processing of the previous emotional experience. More work is required to clarify this further in terms of reflective, mind-wandering or regulatory processing. We have updated our discussion section to better highlight this issue (see page 19).

We thank the reviewer for their really insightful comments and suggestions!

**Reviewer #2 (Public review):**

Summary:

This manuscript examined the neural correlates of the temporal-spatial dynamics of emotional processing while participants were watching short movie clips (each 12.5 s long) from the movie "Forrest Gump". Participants not only watched each film clip, but also gave emotional responses, followed by a brief resting period. Employing fMRI to track the BOLD responses during these stages of emotional processing, the authors found four large-scale brain networks (labeled as IC0,1,2,4) were differentially involved in emotional processing. Overall, this work provides valuable information on the neurodynamics of emotional processing.

Strengths:

This work employs a naturalistic movie watching paradigm to elicit emotional experiences. The authors used a slice-based fMRI method to examine the temporal dynamics of BOLD responses. Compared to previous emotional research that uses static images, this work provides some new data and insights into how the brain supports emotional processing from a temporal dynamics view.

Thank you!

Weaknesses:

Some major conclusions are unwarranted and do not have relevant evidence. For example, the authors seemed to interpret some neuroimaging results to be related to emotion regulation. However, there were no explicit instructions about emotional regulation, and there was no evidence suggesting participants regulated their emotions. How to best interpret the corresponding results thus requires caution.

We thank the reviewer for pointing this out. We have updated the limitations section of our Discussion section (page 20) to better qualify our interpretations.

Relatedly, the authors argued that "In turn, our findings underscore the utility of examining temporal metrics to capture subtle nuances of emotional processing that may remain undetectable using standard static analyses." While this sentence makes sense and is reasonable, it remains unclear how the results here support this argument. In particular, there were only three emotional categories: sad, happy, and fear. These three emotional categories are highly different from each other. Thus, how exactly the temporal metrics captured the "subtle nuances of emotional processing" shall be further elaborated.

This is an important point. We also discuss this limitation in the “limitations” section of our Discussion (page 20). We again thank the reviewer for pointing this out.

The writing also contained many claims about the study's clinical utility. However, the authors did not develop their reasoning nor elaborate on the clinical relevance. While examining emotional processing certainly could have clinical relevance, please unpack the argument and provide more information on how the results obtained here can be used in clinical settings.

We very much appreciate this comment. Note that we did not intend to motivate our study directly from a clinical perspective (because we did not test our approach on a clinical population). Instead, our point is that some researchers (e.g., Kuppens & Verduyn 2017; Waugh et al., 2015) have conceptualized emotional disorders frequently having a temporal component (e.g., dwelling abnormally long on negative thoughts) and that our technique could be used to examine if temporal dynamics of networks are affected in such disorders. However, as we pointed out, this should be verified in future work. We have updated our final paragraph (page 22) to more clearly highlight this issue. We thank the reviewer for pointing this out.

Importantly, how are the temporal dynamics of BOLD responses and subjective feelings related? The authors showed that "the time-to-peak differences in IC2 ("response") align closely with response latency results, with sad trials showing faster response latencies and earlier peak times". Does this mean that people typically experience sad feelings faster than happy or fear? Yet this is inconsistent with ideas such that fear detection is often rapid, while sadness can be more sustained. Understandably, the study uses movie clips, which can be very different from previous work, mostly using static images (e.g., a fearful or a sad face). But the authors shall explicitly discuss what these temporal dynamics mean for subjective feelings.

Excellent point! Our results indeed showed that sad trials had faster reaction times compared to happy and fearful trials, and that this result was reflected in the extracted time-to-peak measures of the fMRI data (see Figure 8D). To us, this primarily demonstrates that, as shown in other studies (e.g., Menon et al., 1997), that gross differences detected in behavioral measures can be directly recovered from temporal measures in fMRI data, which is not trivial. However, we do not think we are allowed to make interpretations of the sort suggested by the reviewer (and to be clear: we do not make such interpretations in the paper). Specifically, the faster reaction times on sad trials likely reflect some audio/visual aspect of the movie clips that result in faster reaction times instead of a generalized temporal difference in the subjective experience of sad vs happy/fearful emotions. Presumably the speed with which emotional stimuli influence the brain depends on the context. Perhaps future studies that examine emotional responses while controlling for the audio/visual experience could shed further light on this issue. We have updated the discussion section to address the reviewer’s concern.

We thank the reviewer for the interesting points which have certainly improved our manuscript!

**Reviewer #1 (Recommendations for the authors):**

Minor:

(1) Please add the unit to the y-axis in Figure 7, if applicable.

Done. We have added units.

(2) Adding a note in the legend of Figure 3 regarding the meaning of the amplitude of the timeseries would be helpful.

Done. We have added a sentence further explaining the meaning of the timecourse fluctuations.

Related references:

(1) Lindquist, M. A., Loh, J. M., Atlas, L. Y., & Wager, T. D. (2009). Modeling the hemodynamic response function in fMRI: efficiency, bias, and mis-modeling. Neuroimage, 45(1), S187-S198.

(2) Méndez-Bértolo, C., Moratti, S., Toledano, R., Lopez-Sosa, F., Martínez-Alvarez, R., Mah, Y. H., ... & Strange, B. A. (2016). A fast pathway for fear in human amygdala. Nature neuroscience, 19(8), 1041-1049.

(3) Bo, K., Cui, L., Yin, S., Hu, Z., Hong, X., Kim, S., ... & Ding, M. (2022). Decoding the temporal dynamics of affective scene processing. NeuroImage, 261, 119532.

(4) Grootswagers, T., Kennedy, B. L., Most, S. B., & Carlson, T. A. (2020). Neural signatures of dynamic emotion constructs in the human brain. Neuropsychologia, 145, 106535.

(5) Gross, J. J. (2015). The extended process model of emotion regulation: Elaborations, applications, and future directions. Psychological inquiry, 26(1), 130-137.
